# A Case Report of Black Hairy Tongue (Melanotrichia Linguae or Lingua Pilosa Nigra)

**DOI:** 10.7759/cureus.60685

**Published:** 2024-05-20

**Authors:** Onur Unal, Ayse Hilal Turker, Fusun Z Akcam

**Affiliations:** 1 Department of Infectious Diseases, Suleyman Demirel University, Isparta, TUR

**Keywords:** pantoprazole, ceftriaxone, anti-bacterial agents, antimicrobial treatment, adverse reactions, drug-related side effect, side effects of medical treatment

## Abstract

Black hairy tongue is a benign condition that can be associated with several varying causes. Its etiology is often linked with fungal infection and adverse reactions to various drugs. We present a case of an adult patient who developed a black hairy tongue while on ceftriaxone and pantoprazole for 10 days. The fungus on his tongue was not identified as the causative agent, and recovery was achieved by changing his medications. Ceftriaxone was replaced with trimethoprim/sulfamethoxazole 5 mg/kg intravenous, and pantoprazole was fully stopped. The black lesion on the tongue was observed to regress over several days. Clinicians should be aware of this particular side effect of certain antibiotics.

## Introduction

Black hairy tongue (lingua villosa nigra) is a benign acquired lesion characterized by hypertrophy, hyperkeratosis, elongation, and carpet-like hairy appearance of the filiform papillae on the dorsal surface of the tongue. Its prevalence is between 0.6-13% in the population, with certain geographical variations [1]. It is frequently asymptomatic and patients mostly present to the physician with aesthetic and appearance-related concerns [2]. In addition to poor oral hygiene, smoking, alcohol, excessive consumption of tea and coffee, and drug use have also been implicated in the etiology of this condition. Among the drugs associated with this condition are antacids, lithium, lansoprazole, and some antibiotics. Among antibiotics, erythromycin, penicillins, doxycycline, linezolid, and neomycin have been most commonly associated with this condition [1-3]. 

The diagnosis is made based on the observation of colored filiform papillae on inspection. A biopsy is typically not required [2]. The differential diagnosis includes agents that color the tongue black, oral hairy leukoplakia, acanthosis nigricans, congenital lingual melanotic macules, congenital melanocytic nevus, premalignant leukoplakia, squamous cell carcinoma, and hypertrophic herpes virus infection [1]. Regarding treatment, it is recommended that patients engage in tongue brushing, take antifungal medications such as oral nystatin, and consume retinoids and B-complex vitamins. Nevertheless, the primary objective is to identify and address the underlying cause.

## Case presentation

**Figure 1:**
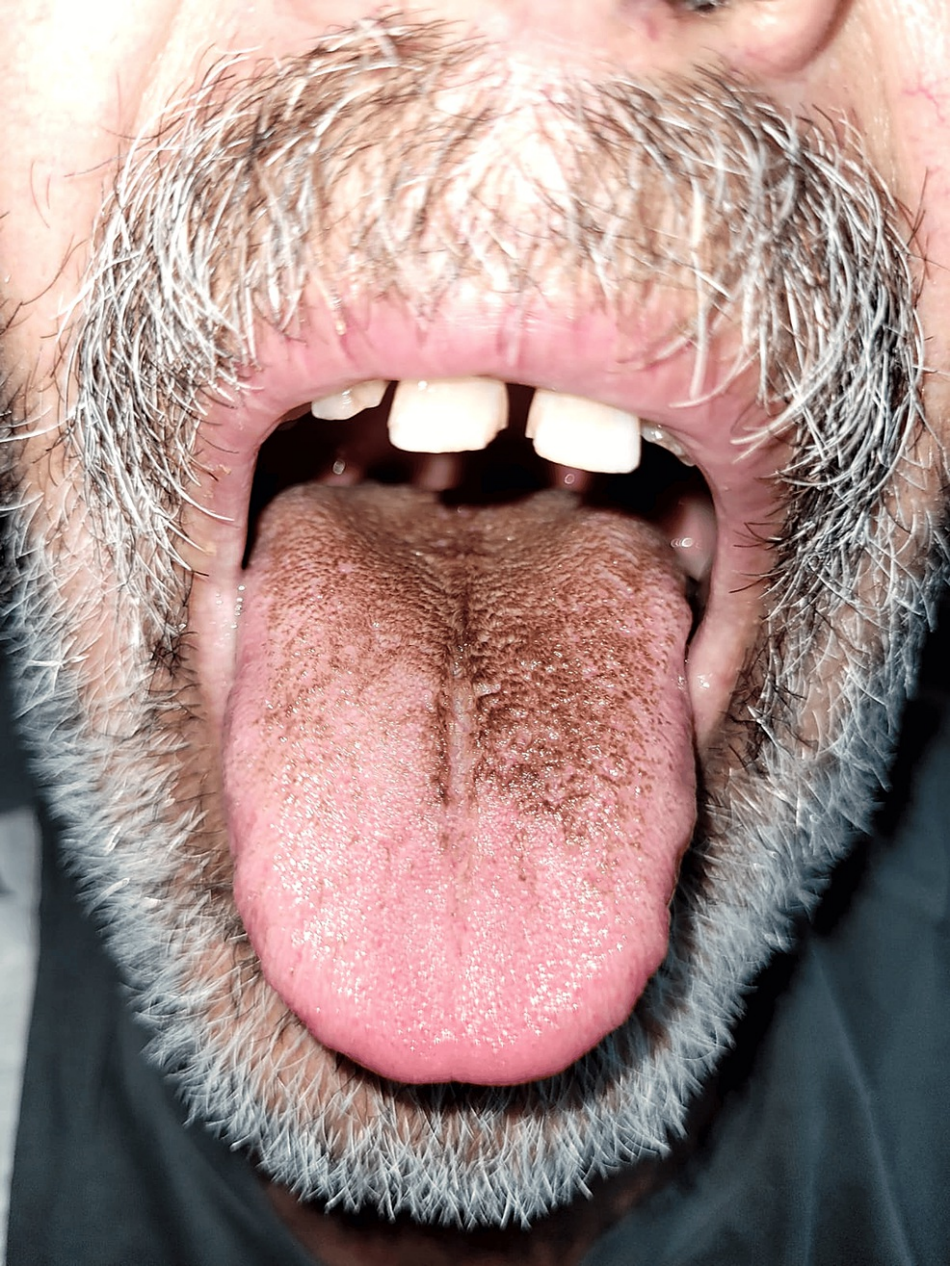
The patient's tongue when a black hairy tongue was first detected

**Figure 2:**
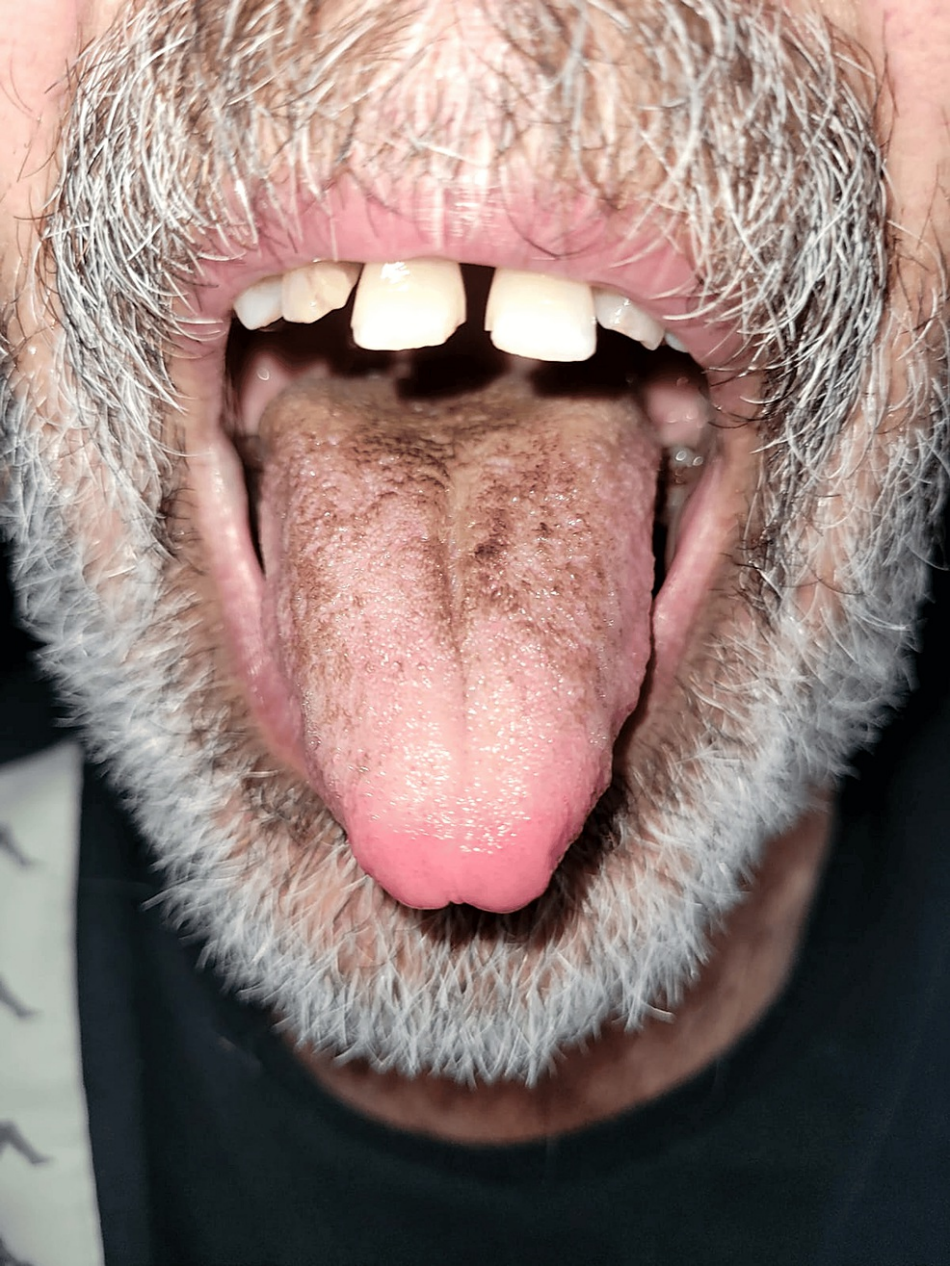
Regression of the lesion on the fifth day of oral maintenance and change in medications

A 64-year-old male patient with a diagnosis of benign prostatic hyperplasia was admitted to the emergency department with complaints of fever and dysuria for three to four days. Non-contrast abdominal CT demonstrated findings consistent with the diagnosis of pyelonephritis in both perirenal areas. Furthermore, microscopic examination of the urine revealed the presence of pyuria. The patient was hospitalized at our clinic with a diagnosis of pyelonephritis and started on parenteral antibiotherapy. Ceftriaxone 2 g twice a day was initiated as empiric treatment. During the hospitalization, urine and blood cultures were obtained, and Escherichia coli was isolated from both. This strain was not producing extended-spectrum beta-lactamase enzymes. Ceftriaxone therapy was continued. On the ninth day of treatment, a new-onset black appearance was observed on the tongue (Figure 1). No fungus was found in the microscopic examination of the lesion. The patient was administered oral care with nystatin and chlorhexidine. The antibiotic treatment was modified to trimethoprim/sulfamethoxazole 5 mg/kg intravenous (IV) on the 10th day and continued for 14 days. The pantoprazole treatment, which was initiated as a stress ulcer prophylaxis, was terminated. Pyelonephritis treatment was completed, and the black lesion on the tongue was observed to have regressed before discharge (Figure 2).

## Discussion

Antibiotics are the most common class of drugs associated with the development of a black hairy tongue. Among antibiotics, penicillin, erythromycin, doxycycline, and neomycin have been most commonly linked to the condition [1]. Our patient had a history of ceftriaxone and pantoprazole use, starting approximately 10 days before the onset of the black hairy tongue. Ceftriaxone is a third-generation cephalosporin antibiotic. Although no case of the black hairy tongue associated with ceftriaxone has been reported in the literature so far, cases associated with the beta-lactam group of antibiotics have been described [4,5]. Furthermore, there are documented cases of black hairy tongue attributed to the use of antacids and proton pump inhibitors [6,7]. It is noteworthy that our literature review revealed cases of hairy tongue that were thought to be of bacterial origin and treated with cefditoren [8] and ceftriaxone [9].

Any discoloration of the tongue may be indicative of an underlying pathological process. The presence of white patches on the tongue may point to several conditions, including thrush, lichen planus, leukoplakia, and others. Red or purple discoloration may indicate harmless conditions such as geographic tongue. However, it can also be suggestive of a vitamin deficiency, scarlet fever, or Kawasaki disease. Yellow discoloration of the tongue is frequently attributed to bacterial overgrowth, dietary habits, or tobacco consumption. In some instances, the presence of a yellow tongue may be indicative of an underlying condition, such as psoriasis or, on rare occasions, jaundice [10]. A dark brown or black discoloration, as observed in our patient, is referred to as a black hairy tongue. Despite the somewhat unusual nomenclature, individuals with black hairy tongues do not have any hair on their tongues. This condition arises when bacteria, food, and other metabolic residues accumulate in the filiform papillae of the tongue [1,10]. The simultaneous presence of more than one of the factors believed to be responsible for the etiology of the black hairy tongue may facilitate the occurrence of the clinical condition.

## Conclusions

Black hairy tongue is a self-limiting, benign disease with a favorable prognosis. Its treatment focuses on mechanical debridement, good oral hygiene, and the removal of potential causative agents. Clinicians need to be aware of the potential link between a black hairy tongue and pantoprazole use in patients, particularly when it is co-administered with antibiotics. We recommend that such cases be documented and reported to create an evidence-based database of similar adverse drug reactions. This report has been prepared with that purpose in mind.
